# Identification of molecular subtypes and a prognostic signature based on m6A/m5C/m1A-related genes in lung adenocarcinoma

**DOI:** 10.1038/s41598-024-57910-5

**Published:** 2024-03-30

**Authors:** Yu Zhang, Qiuye Jia, Fangfang Li, Xuan Luo, Zhiyuan Wang, Xiaofang Wang, Yanghao Wang, Yinglin Zhang, Muye Li, Li Bian

**Affiliations:** 1 Department of Pathology, The First Affiliated Hospital of Kunming Medical University, Kunming, 650302 Yunnan China; 2 Department of Pathology, The Second Affiliated Hospital of Kunming Medical University, Kunming, Yunnan China; 3 Wenshan People’s Hospital, Yunnan, Yunnan Province China

**Keywords:** Lung adenocarcinoma, m6A, m5C, m1A, Signature, Molecule subtypes, Cancer genomics, Cancer models

## Abstract

Lung cancer, specifically the histological subtype lung adenocarcinoma (LUAD), has the highest global occurrence and fatality rate. Extensive research has indicated that RNA alterations encompassing m6A, m5C, and m1A contribute actively to tumorigenesis, drug resistance, and immunotherapy responses in LUAD. Nevertheless, the absence of a dependable predictive model based on m6A/m5C/m1A-associated genes hinders accurately predicting the prognosis of patients diagnosed with LUAD. In this study, we collected patient data from The Cancer Genome Atlas (TCGA) and identified genes related to m6A/m5C/m1A modifications using the GeneCards database. The “ConsensusClusterPlus” R package was used to produce molecular subtypes by utilizing genes relevant to m6A/m5C/m1A identified through differential expression and univariate Cox analyses. An independent prognostic factor was identified by constructing a prognostic signature comprising six genes (*SNHG12*, *PABPC1*, *IGF2BP1*, *FOXM1*, *CBFA2T3*, and *CASC8*). Poor overall survival and elevated expression of human leukocyte antigens and immune checkpoints were correlated with higher risk scores. We examined the associations between the sets of genes regulated by m6A/m5C/m1A and the risk model, as well as the immune cell infiltration, using algorithms such as ESTIMATE, CIBERSORT, TIMER, ssGSEA, and exclusion (TIDE). Moreover, we compared tumor stemness indices (TSIs) by considering the molecular subtypes related to m6A/m5C/m1A and risk signatures. Analyses were performed based on the risk signature, including stratification, somatic mutation analysis, nomogram construction, chemotherapeutic response prediction, and small-molecule drug prediction. In summary, we developed a prognostic signature consisting of six genes that have the potential for prognostication in patients with LUAD and the design of personalized treatments that could provide new versions of personalized management for these patients.

## Introduction

Lung cancer is a fatal malignancy and the leading cause of cancer-related deaths, with an estimated 1.8 million deaths worldwide (almost 18% of all cancers) in 2020^1,2^. Non-small cell lung cancer (NSCLC) and small cell lung cancer (SCLC) are the two major histological types of lung cancer^3^. Lung adenocarcinoma (LUAD), accounting for 40% of all lung cancer cases, is the most prevalent NSCLC subtype^4,5^. Despite substantial progress in treatments, including targeted therapy, surgical treatment, and early cancer detection, the 5-year survival rate of patients with LUAD is approximately 15–20%, and the treatment effectiveness is poor^6,7^. Therefore, exploring new potential biomarkers to predict the clinical prognosis and guide individualized treatment is urgently needed.

RNA methylation, the most stable form of epigenetic modification in RNA posttranscriptional regulation, has been linked to several human diseases^8^. The three major forms of DNA methylation are N6-methyladenosine (m6A), 5-methylcytosine (m5C), and N1-methyladenosine (m1A). Recent studies have found that m6A, m5C, and m1A are involved in tumor regulation by regulating gene expression levels^9–12^. For example, m6A-regulated gene methyltransferase 3 (METTL3) recruits YTHDF1/3 and eIF3b to form a translation initiation complex. This complex plays a crucial role in promoting YAP translation, which in turn contributes to the invasion, metastasis, and chemoresistance of lung cancer cells by mediating miR-1914-3p^13^. m5C methyltransferase NSUN4 is abnormally expressed in LUAD and clear cell renal cell carcinoma and may be utilized to predict prognosis^14,15^. NSUN5 is significantly upregulated in head and neck squamous cell carcinoma (HNSCC) and promotes colorectal cancer (CRC) by inducing cell cycle arrest^16^. m1A is found in mitochondrial transcripts, mRNA, rRNA, and tRNA^17^. m1A demethylase ALKBH3, also known as prostate carcinoma antigen-1 (PCA-1), is not only highly expressed in various human cancers^18^ but also functions as a tRNA demethylase that promotes protein synthesis in cancer cells^19^. Presently, no reliable model based on the m6A, m5C, or m1A genes to predict the prognosis of patients with LUAD exists.

In this study, we developed a prognostic signature based on differentially expressed m6A, m5C, and m1A regulatory genes obtained from The Cancer Genome Atlas (TCGA). We also validated the performance of the signature from the Gene Expression Omnibus (GEO) database; survival stratification, somatic mutations, nomogram construction, chemotherapy response prediction, and small-molecule drug prediction based on risk characteristics were analyzed, thereby providing a reliable prognostic signature.

## Results

### Identification of differentially expressed m6A/m5C/m1A-related gene (DE-MRGs) and biological function analysis

**Figure 1:**
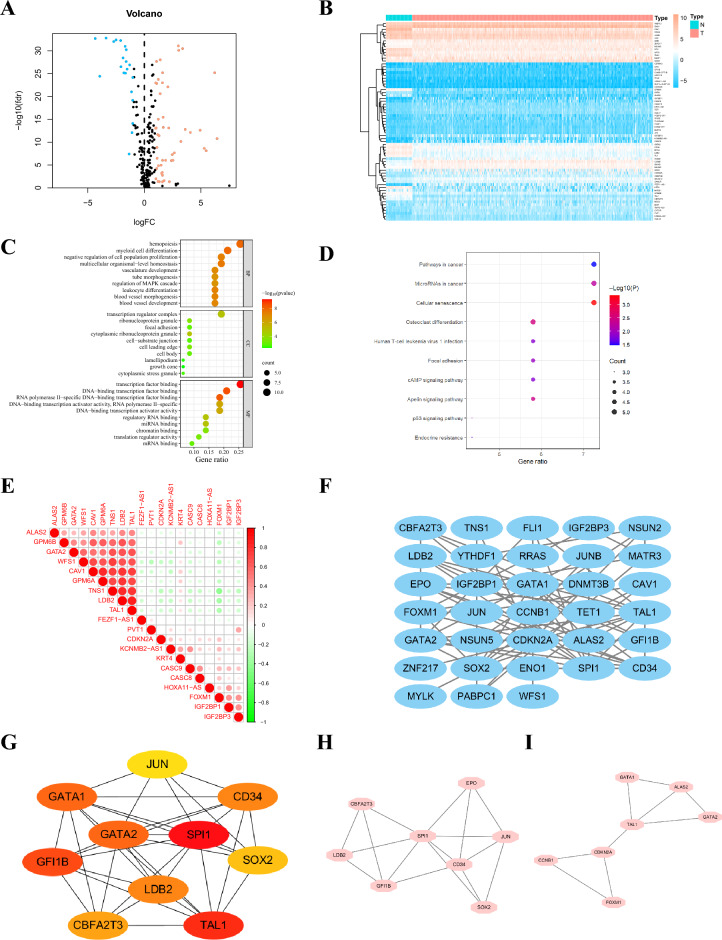
(**A**) Volcano plot of 69 DE-MRGs in LUAD. Red dots represent upregulated genes, and blue dots represent downregulated genes. (**B**) Heatmap of 69 DE-MRGs between normal lung and LUAD tissues. (**C**) The top ten enriched terms in GO analysis for DE-MRGs. (**D**) The top ten enriched terms in KEGG analysis. (**E**) The correlations between the top 10 up-regulated and top 10 down-regulated DE-MRGs. (**F**) PPI network of the DE-MRGs according to the STRING database. (**G**) The top ten hub genes are identified in the PPI network using the “CytoHubba” plugin in Cytoscape. The darker the color, the darker the node degree value. (**H, I**) The two most significant modules are identified from the PPI network using the “MCODE” plugin in Cytoscape. (**H, I**) The hub genes are obtained from the “CytoHubba” plugin in Cytoscape. LUAD, lung adenocarcinoma; DE-MRGs, differentially expressed m6A/m5C/m1A-related genes; GO, gene ontology; BP, biological process; CC, cell component; MF, molecular function; KEGG, Kyoto Encyclopedia of Genes and Genomes; PPI, protein–protein interaction.

Using the GeneCards database and previous research and literature, 64 genes were obtained using relevance scores ≥ 3 as the screening threshold, and 45 genes associated with LUAD were identified, respectively. The study flow is shown in Supplementary Figure S1. Using differential analysis, 48 upregulated genes and 21 downregulated genes in LUAD and normal colon tissues were screened (Fig. 1A, B). Gene ontology (GO) and Kyoto Encyclopedia of Genes and Genomes (KEGG) pathway enrichment analyses revealed that these genes were significantly enriched in the cancer, hemopoiesis, transcription regulator complex, and transcription factor binding pathways (Fig. 1C, D). In addition, correlations between the top ten upregulated and top ten downregulated genes are shown Fig. 1E. The protein–protein interaction (PPI) network was established based on the above differentially expressed genes using the Search Tool for the Retrieval of Interacting Genes (STRING) online database, as shown in Fig. 1F. The node degree represented the degree of connectivity between differentially expressed genes. The Maximal Clique Centrality (MCC) algorithm in the CytoHubba plugin was used to calculate the top ten hub genes in terms of node degree: *SPI1*, *TAL1*, *GFI1B*, *GATA1*, *GATA2*, *LDB2*, *CD34*, CBFA2T3, *SOX2,* and *JUN* (Fig. 1G). Module genes of the PPI analysis were identified using Molecular Complex Detection (MCODE), and the two most significant modules were selected (Fig. 1H, I).

### Identifying m6A/m5C/m1A-associated clusters and correlation analysis between clusters and the tumor immune microenvironment and tumorigenesis scores

**Figure 2:**
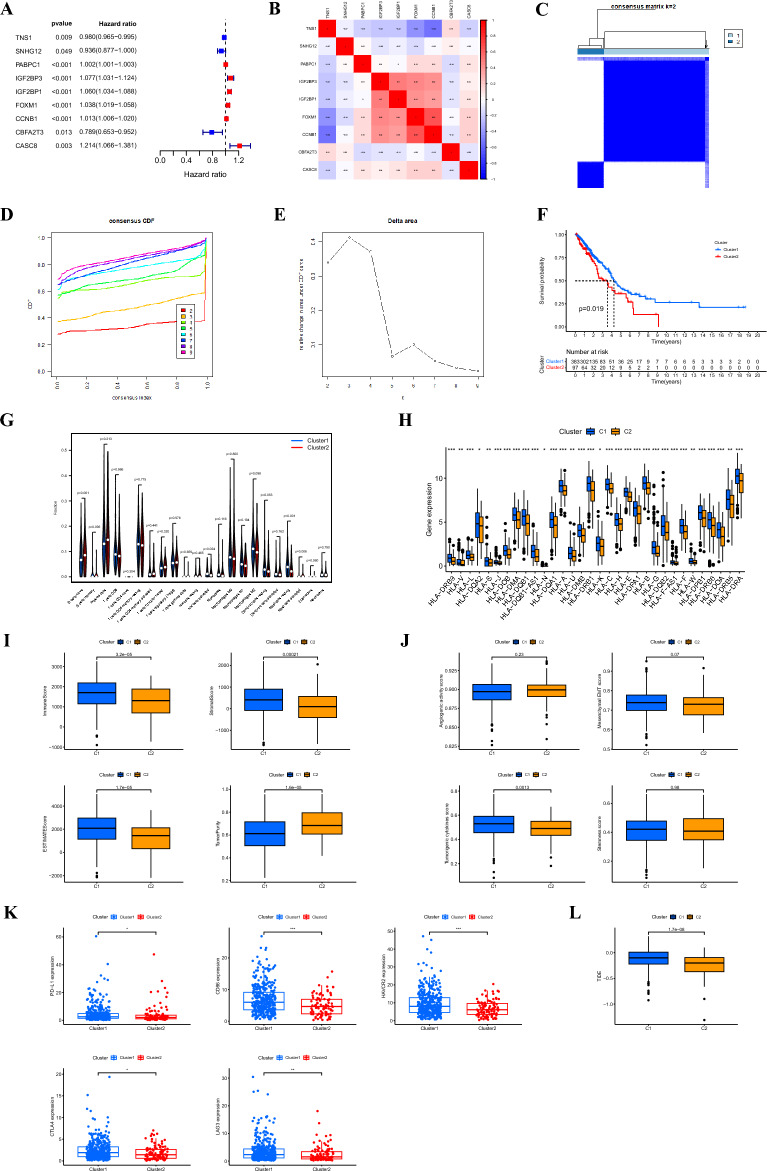
(**A**) Forest plot showing nine prognosis-related DE-MRGs selected by univariate Cox regression analysis. (**B**) Correlations between the nine genes. (**C**) Consensus clustering matrix, when k = 2. (**D**) Consensus clustering CDF with k-values of 2–9. (**E**) Relative change in the area under the CDF curve for k = 2. (**F**) KM curve showing the overall survival in patients between clusters 1 and 2. Immune cell infiltration using CIBERSORT (**G**), the expression of MHC molecules (**H**), immune and stromal scores using ESTIMATE (**I**), angiogenic activity, mesenchymal-EMT, tumorigenic cytokines and stemness scores (**J**), five common immunoinhibitors (**K**), and TIDE scores (**L**) between the two clusters. CDF, cumulative distribution function; KM, Kaplan–Meier; EMT, epithelial-mesenchymal-transition; TIDE, Tumor Immune Dysfunction, and Exclusion. *p < 0.05; **p < 0.01.

Using univariate Cox analysis, we identified nine genes that were associated with prognosis, of which *TNS1*, *SNHG12*, and *CBFA2T3* were protective factors, and the other six genes were risk factors (Fig. 2A, Supplementary Figure S2). The correlation analysis indicated that most genes were correlated with each other (Fig. 2B). Nine prognosis-related genes were used for the cluster analysis. The cluster analysis yielded optimal results when patients with LUAD were divided into two subgroups, and the internal consistency and stability of the subgroups were good (Fig. 2C, E). Survival analysis showed that cluster 1 patients had a better prognosis than cluster 2 patients (Fig. 2F).

Subsequently, the CIBERSORT algorithm was used to analyze the differences in immune infiltration between the two clusters. We found differences in the immune cells between the two clusters (Fig. 2G). The ESITIMATE algorithm showed that cluster 1 had a higher immune score, stromal score, and ESITIMATE score and a lower tumor purity (Fig. 2H). We also found that cluster 1 was related to the higher expression of many MHC molecules (Fig. 2I).

In addition, we examined the angiogenic activity, mesenchymal epithelial-to-mesenchymal transition (EMT), tumorigenic cytokines, and stemness scores between the two groups and found that tumorigenic cytokine scores were significantly higher in cluster 1 (Fig. 2J). We also evaluated their correlation with five common immune checkpoints (*PD-L1*, *CD86*, *HAVCR2*, *CTLA4*, and *LAG3*). Cluster 1 showed a higher expression of all immune checkpoints (Fig. 2K) and was associated with higher TIDE scores (Fig. 2L).

### Construction of a m6A/m5C/m^1^A-related signature and nomogram based on the signature

**Figure 3:**
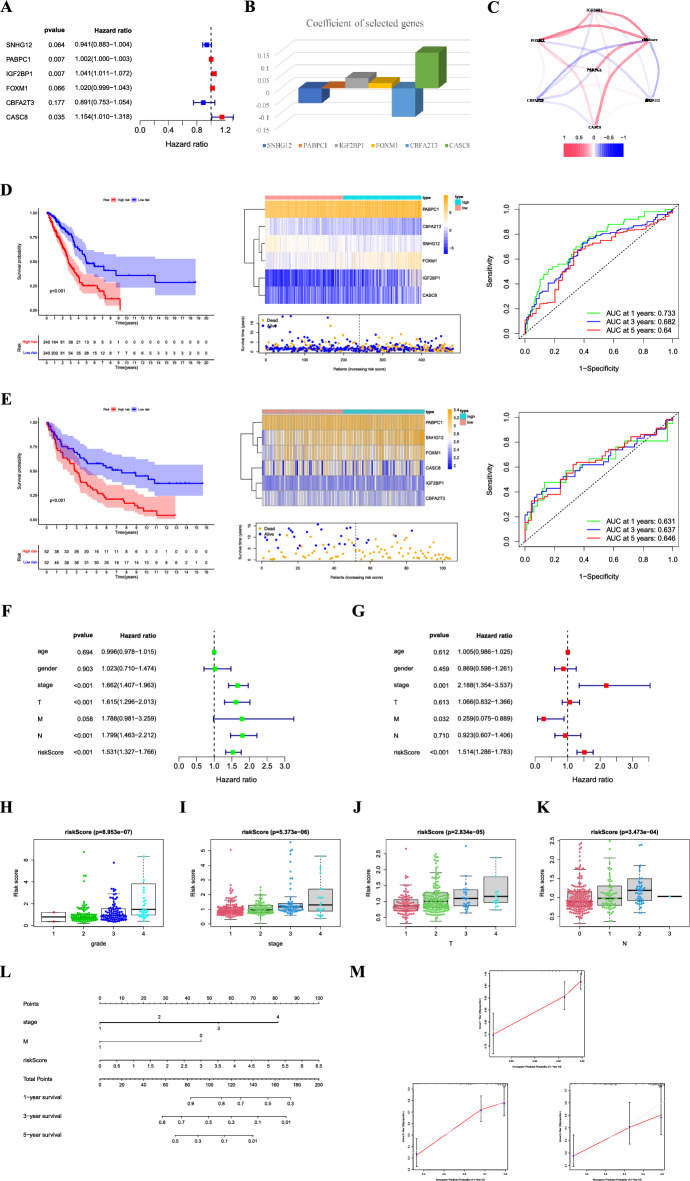
(**A**) Forest plot showing six genes selected in the signature through multivariate Cox analysis. (**B**) Coefficients of the four genes included in the signature. (**C**) Correlations between the signature and the six genes. KM survival analysis, heatmap, survival status accompanied with the risk score, and ROC analysis in the TCGA cohort (**D**) and GSE37745 cohort (**E**). Univariate (**F**) and multivariate Cox analyses (**G**) show that signature is an independent risk factor for patients with LUAD in the TCGA cohort. Risk score differences between groups according to clinicopathological features, including the grade (**H**), stage (**I**), T stage (**J**), and N stage (**K**). (**L**) Nomogram combining clinicopathological variables and risk scores to predict overall survival at 1, 3, and 5 years of patients with LUAD. (**M**) The calibration curves of the nomogram for predicting the probability of 1-, 3-, and 5-year survival. ROC, receiver operating characteristic; TCGA, The Cancer Genome Atlas; M, metastasis.

To further screen the genes included in the model, we performed a multifactor Cox regression analysis and identified six genes for inclusion in the signature (Fig. 3A). The coefficients for each gene in the signature are shown in Fig. 3B. Figure 3C shows the correlation between the risk score and *SNHG12*, *PABPC1*, *IGF2BP1*, *FOXM1*, *CBFA2T3*, and *CASC8*. Risk scores were calculated as follows:

$$\begin{aligned} Risk\,score\, = & \,\left( { - \,0.0{6}0{5}\, \times \,SNHG12\,{\text{expression}}} \right)\, + \,\left( {0.00{18}\, \times \,PABPC1\,{\text{expression}}} \right) \\ & + \,\left( {0.0{4}0{3}\, \times \,IGF2BP1\,{\text{expression}}} \right)\, + \,\left( {0.0{2}0{2}\, \times \,FOXM1\,{\text{expression}}} \right) \\ & + \,\left( { - \,0.{1154}\, \times \,CBFA2T3\,{\text{expression}}} \right)\, + \,\left( {0.{1431}\, \times \,CASC8\,{\text{expression}}} \right) \\ \end{aligned}$$

Patients with high-risk scores had a poorer prognosis than those with low-risk scores, and the area under the curve (AUC) of the signature was 0.733 at one year (Fig. 3D). The GEO dataset was used to validate the efficiency of the signature, which showed good efficiency (Fig. 3E). Stratified analysis revealed that this signature could effectively differentiate the patient prognosis across various clinical subgroups. Specifically, patients classified in the high-risk group exhibited a notably poorer prognosis (Supplementary Figure S3). In addition, univariate and multivariate Cox regression analyses indicated that the risk signature was an independent risk factor for LUAD (Fig. 3F, G). We also analyzed the differences in risk scores between subgroups based on different clinicopathological parameters. The results indicated that patients with grade III–IV, stage III–IV, T3–4, and N3 disease had higher risk scores. This implied that the tumors became more advanced as the risk score increased (Figs. 3H–K).

To enhance the ability to predict the prognosis of patients with LUAD, we constructed a nomogram incorporating clinicopathological variables and risk scores to predict the prognosis of patients with ccRCC at 1, 3, and 5 years (Fig. 3L). The calibration curve showed excellent agreement between the actual overall survival (OS) and the predicted survival at 1, 3, and 5 years (Fig. 3M).

### Estimation of tumor immune cell infiltration and immune checkpoint inhibitors according to the signature

**Figure 4:**
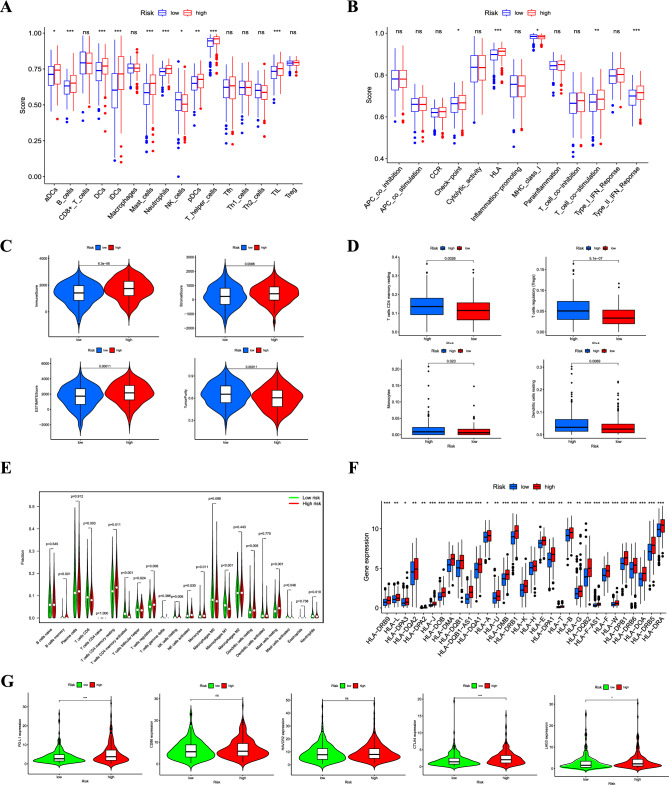
(**A**) Infiltration levels of 16 immune cells in the high- and low-risk groups using the ssGSEA algorithm. (**B**) Correlation of predictive signature with 13 immune-related functions or pathways. Immune and stromal scores (**C**), immune cell infiltration using TIMER (**D**) and CIBERSORT (**E**), MHC molecule expression levels (**F**)**,** and five common immunoinhibitors (**G**) between the high- and low-risk groups. (*p < 0.05; **p < 0.01; ***p < 0.001; ns, not significant).

Considering the varying prognoses of patients in the high- and low-risk groups, we performed gene set enrichment analysis (GSEA). The intestinal immune network for immunoglobulin A production and pathways for primary immunodeficiency were significantly enriched in the high-risk group (Table S1), suggesting that a high risk is closely associated with an immune response. Therefore, we further investigated the relationship between this signature and the tumor immune microenvironment. The single-sample Gene Set Enrichment Analysis (ssGSEA) algorithm revealed that the high-risk group exhibited a greater degree of immune cell infiltration and a higher number of immune-related functions or pathways than the low-risk group (Fig. 4A, B). Using the ESTIMATE algorithm, patients in the high-risk group had higher immune, stromal, and estimation scores and lower tumor purity (Fig. 4C). Immune cells that predominantly infiltrated the high-risk group included memory CD4 + T cells, regulatory T cells, monocytes, and resting dendritic cells (Fig. 4D). Memory B cells, CD8 + T cells, and resting mast cells were also higher in the high-risk group (Fig. 4E). Additionally, we detected the expression of MHC molecules and found that the low-risk group exhibited significantly lower expression levels (Fig. 4F). We also evaluated their correlation with five common immune checkpoints (*PD-L1*, *CD86*, *HAVCR2*, *CTLA4*, and *LAG3*). In the high-risk group, immune checkpoints *PD-L1*, *CTLA4*, and *LAG3* showed higher expression (Fig. 4G).

### Correlation of angiogenic activity, mesenchymal EMT, tumorigenic cytokines, stemness scores and TSIs with the signature

**Figure 5:**
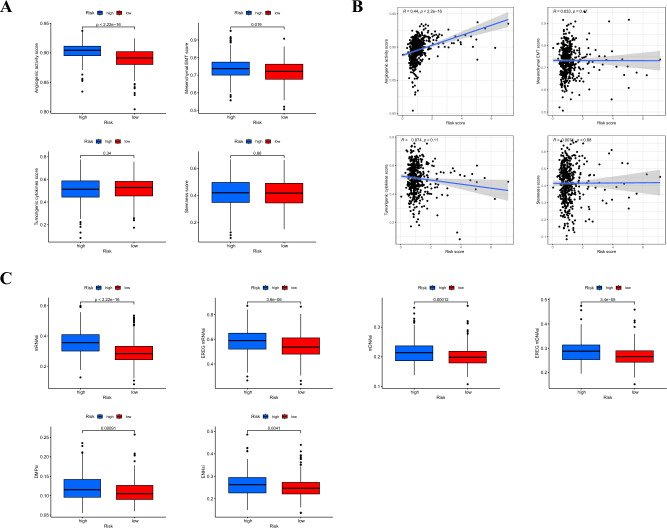
(**A**) Differences in angiogenic activity, mesenchymal EMT, tumorigenic cytokines, and stemness scores between the high- and low-risk groups. (**B**) Correlation of the risk score and the angiogenic activity, mesenchymal EMT, tumorigenic cytokines, and stemness scores. (C) Differences in TSIs between the two groups. TSIs, tumor stemness indices. (*p < 0.05; **p < 0.01; ***p < 0.001; ns, not significant).

Previous studies identified clusters linked to angiogenic activity, mesenchymal EMT, tumorigenic cytokines, and stemness scores. Therefore, our objective was to investigate whether these four tumor-associated functions play a role in the underlying mechanisms of the signature. We calculated the angiogenic activity, mesenchymal EMT, tumorigenic cytokines, and stemness scores in patients with LUAD. The high-risk group showed higher angiogenic activity and mesenchymal EMT (Fig. 5A). Correlation analysis revealed that the risk score was significantly and positively associated with angiogenic activity (R = 0.44, *p* = 2.2e-16) (Fig. 5B). In addition, the low-risk group exhibited lower levels of TSIs, including mRNAsi, EREG-mRNAsi, mDNAsi, EREG-mDNAsi, and ENHsi (Fig. 5C).

### Comparison of somatic mutation and tumor mutation burden (TMB) in the signature

**Figure 6:**
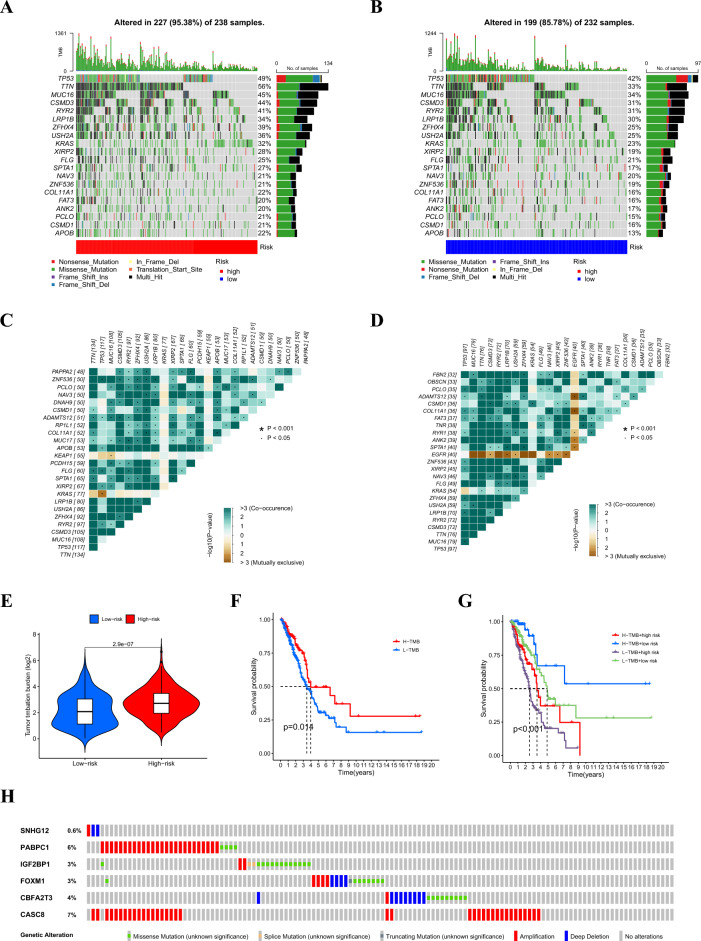
Waterfall plot of somatic mutation features in the high-risk group (**A**) and low-risk group (**B**). Heatmap of co-occurrence and mutually exclusive mutations of the differently mutated genes in the high-risk group (**C**) and the low-risk group (**D**). (**E**) Comparison of the difference in TMB between high- and low-risk groups. (**F**) The difference in overall survival between high- and low-TMB groups. (**G**) The difference in overall survival is based on the TMB and risk score. (**H**) Mutation rates of six genes (*SNHG12*, *PABPC1*, *IGF2BP1*, *FOXM1*, *CBFA2T3*, and *CASC8*) in patients with LUAD from the cBioPortal database.

To investigate genomic mutations between the high- and low-risk groups, we obtained simple nucleotide variation data from the TCGA. In the high-risk group, the five genes with the highest mutation frequencies were *TTN* (56%), *TP53* (49%), *MUC16* (45%), *CSMD3* (44%), and *RYR2* (41%). In the low-risk group, the five genes with the lowest mutation frequencies were *TP53* (42%), *TTN* (33%), *MUC16* (34%), *CSMD3* (31%), and *RYR2* (31%) (Fig. 6A, B). In addition, we identified somatic mutation interactions and observed gene mutation co-occurrence among most genes. Notably, the high-risk group showed mutually exclusive *TP53*-*KRAS* mutations (Fig. 6C). Although gene mutation co-occurrence was common in low-risk groups, mutually exclusive mutations existed among a significant number of genes (Fig. 6D). Further correlation analysis suggested that the TMB of patients with LUAD in the high-risk group was significantly higher than that of patients in the low-risk group (Fig. 6E). There was no difference in survival between the patients with high and low TMB (Fig. 6F). By combining the TMB with the risk model, we found that patients in the low-risk and high-TMB group had the best prognosis, whereas those in the high-risk and low-TMB group had the worst (Fig. 6G). Finally, we detected the mutation rates of six genes in the signature and found that they were low (Fig. 6H).

### Small-molecule drug screening

**Figure 7:**
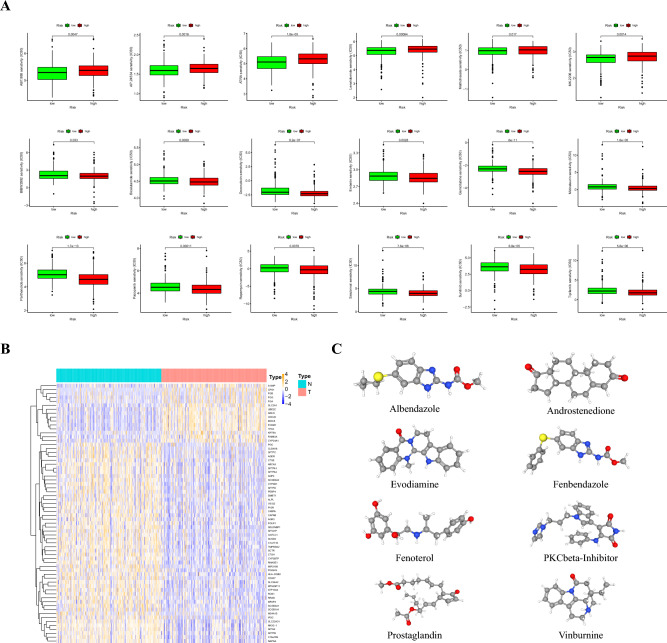
(**A**) Comparison of common chemotherapy drug sensitivities between high- and low-risk groups. (**B**) Differentially expressed genes between the high- and low-risk groups. (**C**) The 3D structure of eight potential target drugs screened from the cMap database.

To further explore individualized treatment regimens, we calculated the half-maximal inhibitory concentration (IC50) of 95 chemotherapy drugs in the high- and low-risk groups based on LUAD data from the TCGA. BIBW2992, bicalutamide, doxorubicin, embelin, gemcitabine, midostaurin, parthenolide, pazopanib, rapamycin, salubrinal, sunitinib, and tipifarnib were candidate drugs for treating high-risk patients (Fig. 7A). We identified 15 upregulated and 51 downregulated genes by comparing the low- and high-risk groups, respectively (Fig. 7B). Subsequently, we used the cMap database to screen for small-molecule drugs. The eight most relevant drugs were screened as prospective candidates for treating patients with LUAD based on the differentially expressed genes. The 3D structures of albendazole, androstenedione, evodiamine, fenbendazole, fenoterol, prostaglandin PKCβ-inhibitor, and vinburnine are shown in the PubChem database (Fig. 7C).

## Discussion

According to recent studies, LUAD has emerged as the predominant form of NSCLC, surpassing lung squamous cell carcinoma^20^. The 5-year survival rate for this type of cancer is only 15%^21^. In recent years, an increasing number of studies have focused on the effects of RNA methylation on the occurrence, development, and prognosis of LUAD.

In the present study, we identified 109 DE-MRGs between tumor and normal tissues based on LUAD data from the TCGA. We conducted a systematic analysis of relevant biological pathways and constructed co-expression and PPI networks. Moreover, we performed several statistical analyses to determine the predictive capabilities of a signature comprising the six DE-MRGs in patients with LUAD. These analyses included univariate Cox regression, survival, multiple stepwise Cox regression, and receiver operating characteristic (ROC) analyses. Our results showed that a signature consisting of *SNHG12*, *PABPC1*, *IGF2BP1*, *FOXM1*, *CBFA2T3*, and *CASC8* could accurately predict clinical outcomes and treatment responses in patients with LUAD.

*SNHG12* is a small nucleolar RNA host gene (SNHG) implicated in cancer progression^22^. Ruan et al. demonstrated that *SNHG12* plays a role in cell proliferation and migration^23^. Previous studies have reported *SNHG12* overexpression in CRC and breast cancer^24,25^. *IGF2BP1*, a member of the IGF2BP family, is an m6A-binding protein that recognizes GG(m6A)C sequences within targeted mRNA, thereby participating in the transcription, stability, splicing, and translation of various RNA molecules^26^. Previous studies demonstrated that *IGF2BP1* interacts with LIN28B-AS1 to enhance the stability and translation of mRNA in a manner influenced by m6A modification, which ultimately leads to increased proliferation and invasion of cancer cells^27–29^. Previous studies have shown that *FOXM1* is important for regulating various processes involved in lung cancer tumorigenesis, including cell cycle progression, cancer therapy resistance, and metastasis^30–32^. For example, *FOXM1* plays a role in regulating the radiosensitivity of lung cancer cells, partially through the upregulation of *KIF20A*^33^, and *FOXM1* activation leads to the progression of lung adenomas into invasive mucinous adenocarcinomas by activating *AGR2*^34^. *CBFA2T3*, a chromatin repressor localized in the nucleolus, functions as a suppressor of breast tumorigenesis^35^. *CBFA2T3* is also a tumor suppressor in lung cancer and can serve as an independent prognostic marker for LUAD^36^. Given the positive correlation between *CBFA2T3* expression and higher antigen-presenting cell infiltration, *CBFA2T3* holds promise as a potential tumor antigen for future mRNA vaccines^37^. Studies have shown that cancer susceptibility candidate 8 (*CASC8*) is a tumor susceptibility gene^38,39^. Interestingly, *CASC8* regulates EMT genes via *FOXM1*. Moreover, *CASC8* suppression substantially decreases the proliferation, migration, and invasion of NSCLC cells^38^.

Further analyses showed the high predictive accuracy and robustness of the m6A/m5C/m1A-related gene signature in this study. Moreover, the patients in the high-risk group had poorer outcomes in the training and validation cohorts. The 3-year survival AUC was 0.733 in the TCGA cohort and 0.631 in the GSE37745 cohort. Univariate and multivariate Cox regression analyses identified the risk score as an independent prognostic factor. Subsequently, we developed a quantitative and objective nomogram based on multivariate analysis. Therefore, our nomogram is suitable for clinical practice.

The tumor microenvironment has garnered considerable attention because of its substantial influence on cancer progression, including tumor growth, invasion, and metastasis^40,41^. In this study, the m6A/m5C/m1A-related signature was significantly associated with immune cell infiltration. For example, the high-risk group was infiltrated by a higher proportion of memory B cells, CD8 + T cells, resting memory CD4 + T cells, regulatory T cells, monocytes, resting dendritic cells, and resting mast cells. CD8 + T cells are essential for establishing antitumor immunity within the tumor microenvironment of LUAD. These cells play a significant role in antitumor immunity, and CD4 + T cells contribute to this antitumor effect by secreting various cytokines and assisting in activating CD8 + T and other immune cells.

Immune checkpoints are molecules expressed by immune cells sensitive to the regulation of immune activation^42^. We investigated the expression of five immune checkpoint genes in the two risk subtypes in this study. These findings revealed increased expression of the three immune checkpoint genes in high-risk patients. This suggests that patients in the high-risk group may benefit from immunotherapies that inhibit immune checkpoints.

Resistance to chemotherapy is a significant challenge encountered during LUAD treatment that contributes to the high mortality rate associated with cancer. Identifying a chemosensitive population may enhance the antitumor effects in patients with LUAD who can benefit from standard chemotherapeutic regimens.

Patients in the high-risk group were sensitive to ABT.888, AICAR, MS.275, sunitinib, AZD.2281, and GDC.0449. AT.888 has been used to treat a wide variety of tumors. Patients in the high-risk group were sensitive to BIBW2992, bicalutamide, doxorubicin, embelin, gemcitabine, midostaurin, parthenolide, pazopanib, rapamycin, salubrinal, sunitinib, and tipifarnib, which can be used to treat a wide range of tumors. Bicalutamide, a second-generation oral nonsteroidal antiandrogen, is widely used to treat prostate cancer and triple-negative breast cancer^43,44^. Sunitinib is a tyrosine kinase inhibitor with antiangiogenic properties that plays a crucial role in the treatment of metastatic renal cell carcinoma^45^. We also used the cMap database to screen eight small-molecule drugs for LUAD treatment. Therefore, the results of this study can be used to provide personalized and precise drug treatment for high-risk patients.

Our study had several limitations. First, we conducted a retrospective analysis of the signature using data from the GEO database. However, to further assess its clinical value, it is imperative to conduct more prospective studies. Second, further in vivo and in vitro investigations are necessary to explore the involvement of the six selected genes in the development of LUAD. Third, this study analyzed the correlation between risk models and various aspects of the immune system, including immune cells, immune function, MHC molecules, immune checkpoints, and immunotherapy. However, sufficient samples to assess the efficacy of this model in conjunction with immunotherapy were not collected. Furthermore, a more thorough evaluation of the model by incorporating additional metrics, such as the variation statistics, unbiased concordance statistic K, and the Royston-Sauerbrei D statistic^46^, would be valuable.

In conclusion, we developed a novel m6A/m5C/m1A-related gene signature that can effectively predict the prognosis of patients with LUAD. This study establishes a foundation and highlights the potential predictive significance of the mechanisms connecting m6A/m5C/m1A-related genes to LUAD. Our findings may contribute to the development of novel clinical interventions for this disease.

## Materials and methods

### Data source

The fragments per kilobase of transcript per million mapped reads (FPKM)-normalized transcript RNA-sequencing data, relevant clinical information, and simple nucleotide variation data were downloaded from the TCGA database (https://portal.gdc.cancer.gov). The GSE37745 dataset was downloaded from the GEO database to validate the signature. The list of m6A/m5C/m1A-related genes was obtained from the GeneCards database (https://www.genecards.org), previous studies, and literature^10,47–49^.

### Analysis of DE-MRGs

m6A/m5C/m1A-related genes were obtained by comparing 59 normal and 541 LUAD tissues in the TCGA using the following criteria: |log fold change (FC)|> 1 and a false discovery rate (FDR) < 0.05. The genes were analyzed using Metascape (https://metascape.org)^50^ for GO and KEGG analyses and visualized using ggplot2.

### Hub DE-MRGs selection and analyses

The online STRING database (https://string-db.org/) was used to analyze the interactive relationships among the DE-ERGs. Parameter settings were as follows: network scoring, degree cutoff = 2 and cluster finding, node score cutoff = 0.2, k-core = 2, and maximum depth = 100. Cytoscape software (version 3.7.0) was used to create PPI networks. The top ten DE-MRGs with higher node degree scores were identified as hub ERGs using the Cytoscape plug-in CytoHubba. Hub modules were analyzed using MCODE plug-ins in Cytoscape.

### Cluster analysis

Prognosis-related DE-MRGs were screened using univariate Cox regression analysis. The “ConsensusClusterPlus” package was used to perform cluster analysis to identify m6A/m5C/m1A-related molecule subtypes^51^. Survival analysis was performed to compare the prognosis between the two clusters. A heatmap was used to display the correlation between clusters and clinical parameters, which were analyzed using the chi-square test.

### Construction and validation of the prognostic signature

Multivariate Cox regression analysis was used to identify DE-MRGs that provided a prognostic signature. ROC and KM analysis were used to evaluate the prognostic value of the signature^52,53^. The GSE37745 dataset was used to validate the prognostic signatures. Univariate and multivariate Cox regression analyses were performed to determine whether the signature was an independent risk factor. We combined risk scores with clinicopathological characteristics to construct a nomogram that predicted survival at 1, 3, and 5 years in patients with LUAD. Calibration curves were used to determine whether the predicted survival rates were consistent with actual survival rates.

### GSEA

Patients with LUAD were divided into high- and low-risk groups based on the median risk scores. To explore the potential underlying mechanisms, we used GSEA v4.1.0 (http://www.broad.mit.edu/gsea) to investigate the enriched pathways in the high-risk group, with *p* < 0.05 and FDR < 0.25 as thresholds for statistical significance^54^.

### Immune landscape analysis

The infiltration scores of 16 immune cells and the activities of 13 immune-related pathways were calculated using the GSVA software package employing the ssGSEA method^55^. Marker genes of various immune cells were obtained from previous studies^56^ and are listed in Table S2. The ESTIMATE algorithm was used to calculate the immune score, stromal score, estimated score, and tumor purity of all patients with LUAD. Immune cell infiltration was analyzed using the CIBERSORT algorithm and TIMER database. We also compared the expression of MHC molecules using cluster and signature analyses.

We compared five commonly studied immunoinhibitors (*PD-L1*, *CD86*, *HAVCR2*, *CTLA4*, and *LAG3*) based on clusters and risks for the immune checkpoints.

### Tumor-related scores and tumor stemness indices (TSIs) analysis

The ssGSEA algorithm was utilized to calculate the scores of angiogenic activity, mesenchymal-EMT, tumorigenic cytokines, and stemness for each tumor sample, and relevant marker genes are listed in Table S3. TSIs were closely linked to active biological processes in stem cells and correlated with a higher degree of tumor dedifferentiation. We obtained TSIs from the TCGA in a previous study^57^.

### Gene mutation analysis

Somatic mutation expression data were obtained from the TCGA and analyzed using the R package “maftools.” We calculated the TMB for each patient and compared it between the high- and low-risk groups. Additionally, we conducted a survival analysis based on the TMB scores. The cBioPortal database was used to display somatic mutations in selected genes in the signature^58^.

### Chemotherapy response and small-molecule drugs

To investigate the predictive role of the signature concerning the clinical response to treatment, we utilized the 'pRRophetic' package to calculate the IC50 values of commonly used chemotherapeutic agents for the clinical treatment of LUAD. The Wilcoxon signed-rank test was used to compare the IC50 values of the chemotherapeutic drugs between the high- and low-risk groups.

### Statistical analysis

DE-MRGs were screened using the Wilcoxon test. Univariate Cox regression analysis was used to analyze the relationship between m6A/m5C/m1A-associated genes and overall survival (OS). Multifactorial Cox regression analysis was used to screen for genes associated with m6A/m5C/m1A and construct a predictive signature. Kaplan–Meier survival analysis and log-rank tests were used to analyze the differences in OS between the different risk score groups. The "timeROC" package was utilized to generate ROC curves and calculate the AUC. All statistical analyses were performed using R software v4.1.3 and its appropriate websites^59^.

### Supplementary Information

Supplementary Figure S1.

Supplementary Figure S2.

Supplementary Figure S3.

Supplementary Legends.

Supplementary Table S1.

Supplementary Table S2.

Supplementary Table S3.

## Data Availability

Some or all data, models, or code generated or used during the study are available from the corresponding author by request.
