# Supplementary figures and images for: Identification of molecular subtypes and a prognostic signature based on m6A/m5C/m1A-related genes in lung adenocarcinoma
Source: Sci Rep. 2024 Mar 30;14:7543. doi: 10.1038/s41598-024-57910-5 (PMC10981664; doi:10.1038/s41598-024-57910-5)

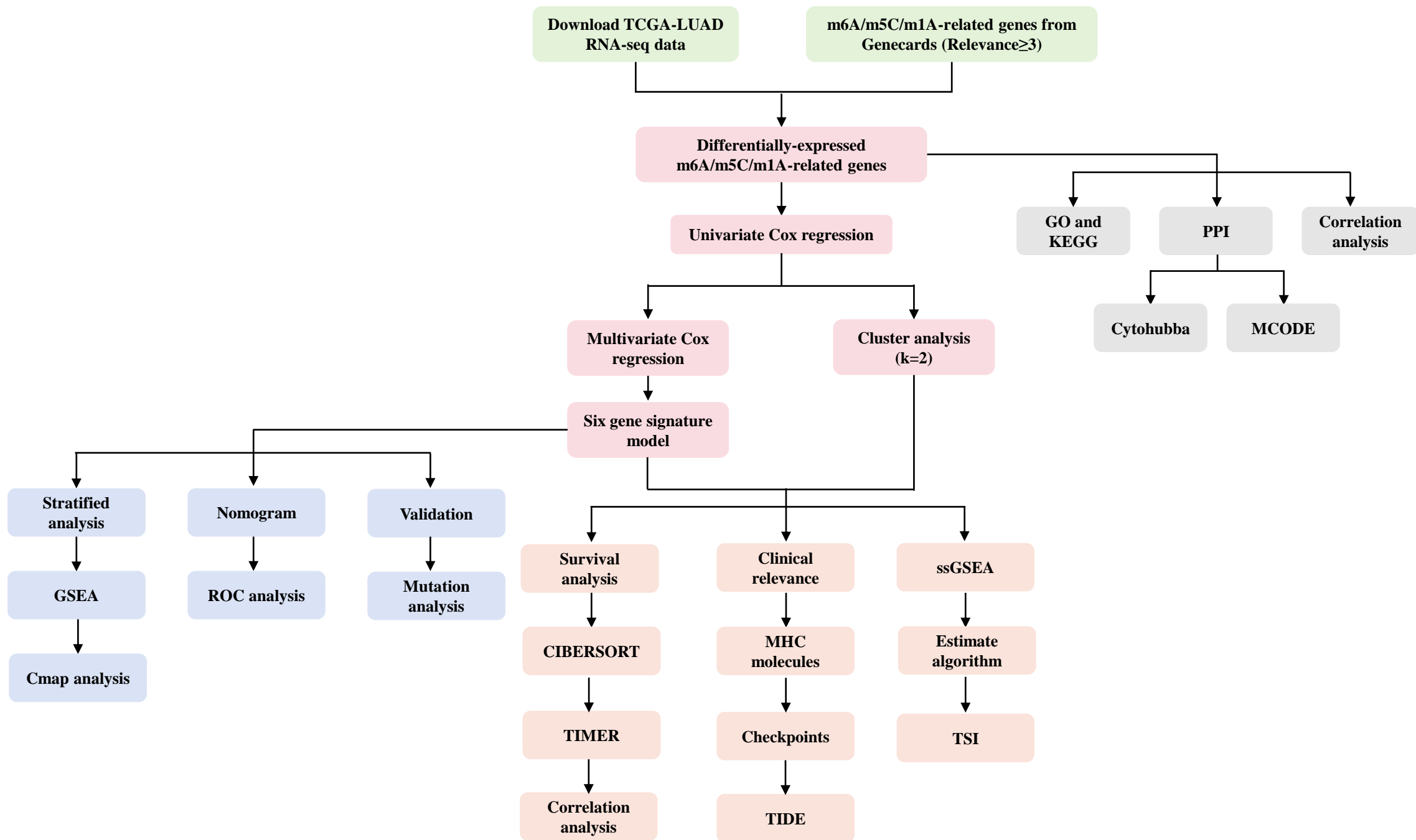

Supplement: Supplementary file 1 — Supplementary Figure S1. [file 41598_2024_57910_MOESM1_ESM.pdf]

**A****TNS1**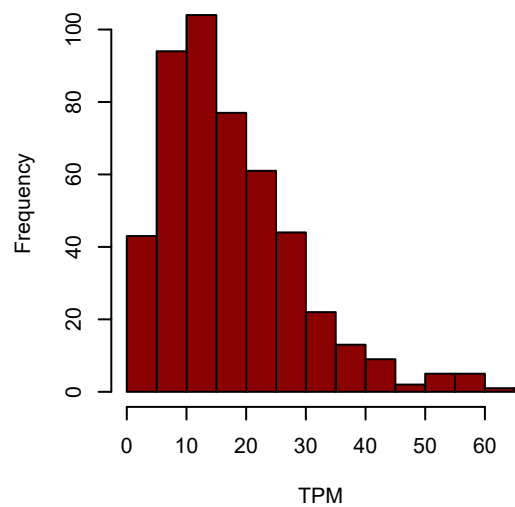**B****SNHG12**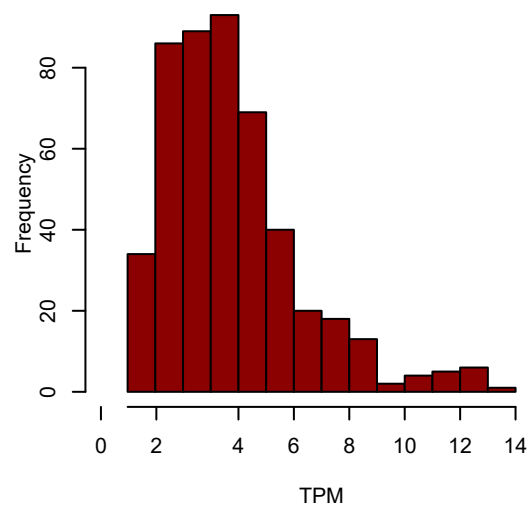**C****PABPC1**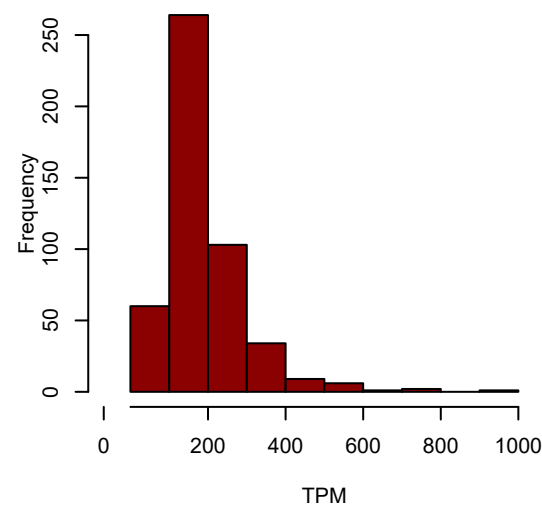**D****IGF2BP1**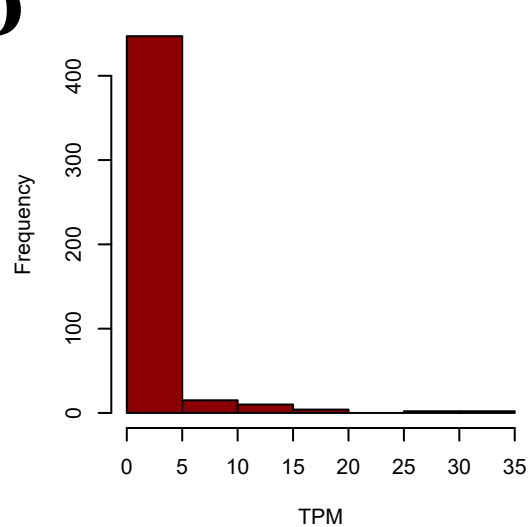**E****FOXM1**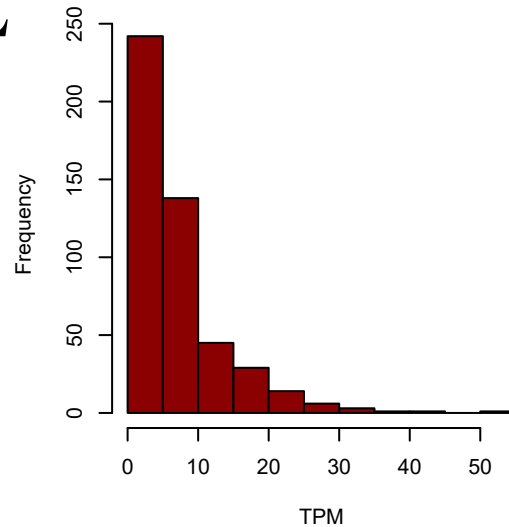**F****CCNB1**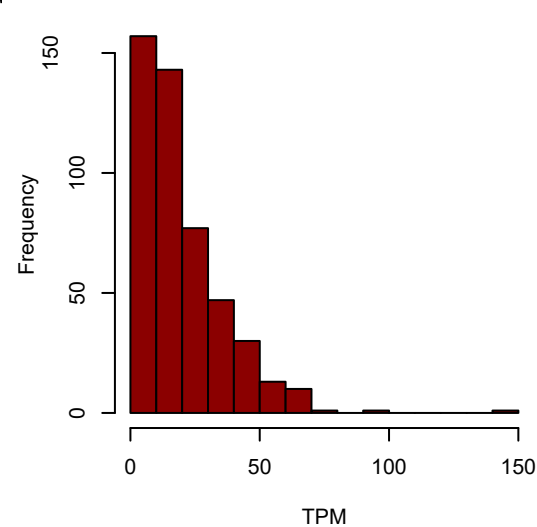**G****IGF2BP3**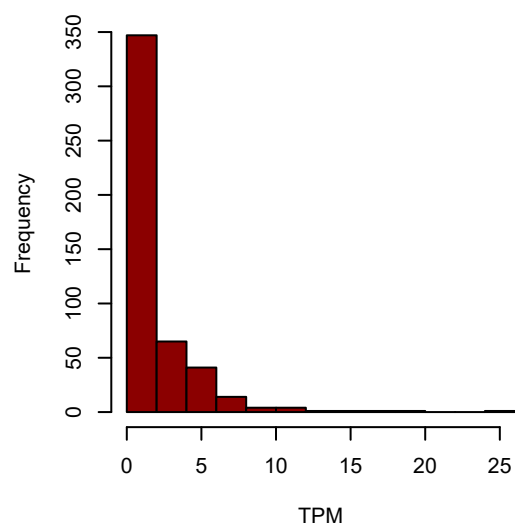**H****CBFA2T3**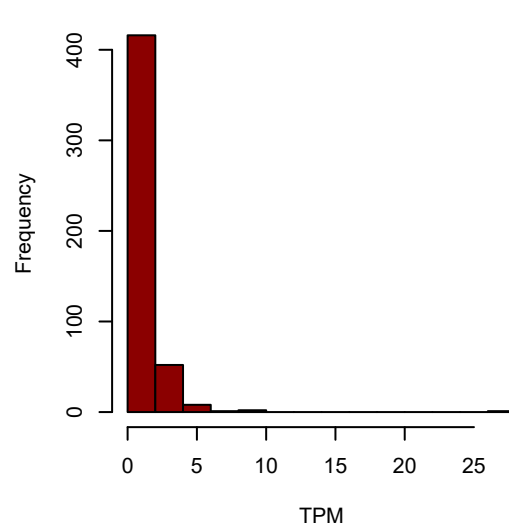**I****CASC8**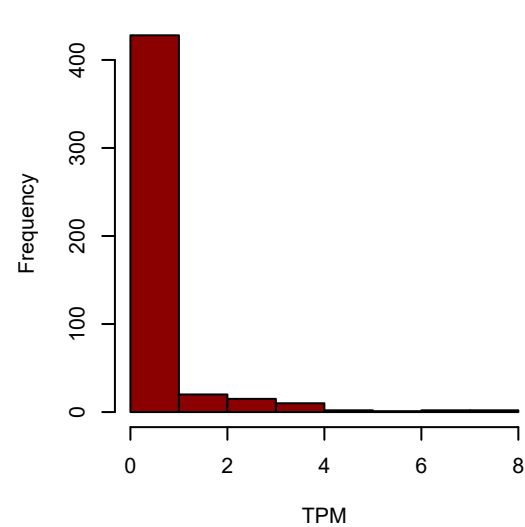

Supplement: Supplementary file 2 — Supplementary Figure S2. [file 41598_2024_57910_MOESM2_ESM.pdf]

A

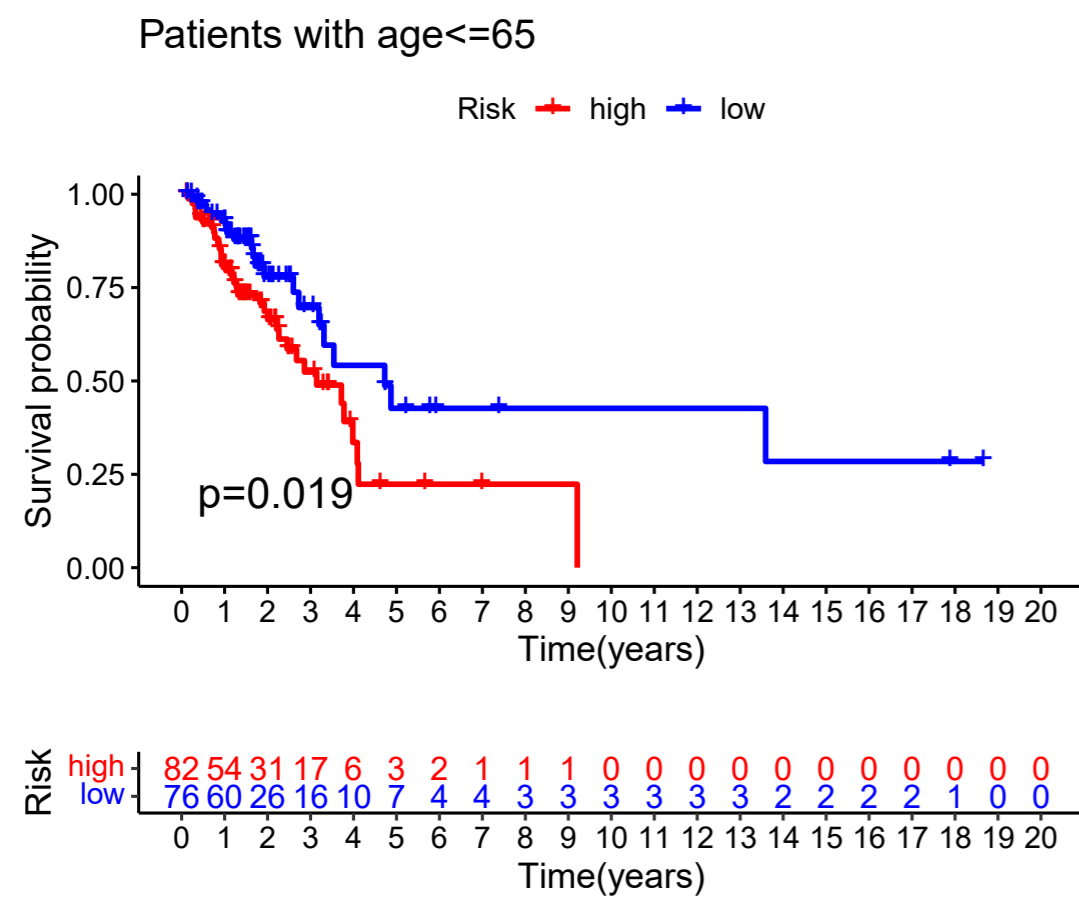

# B

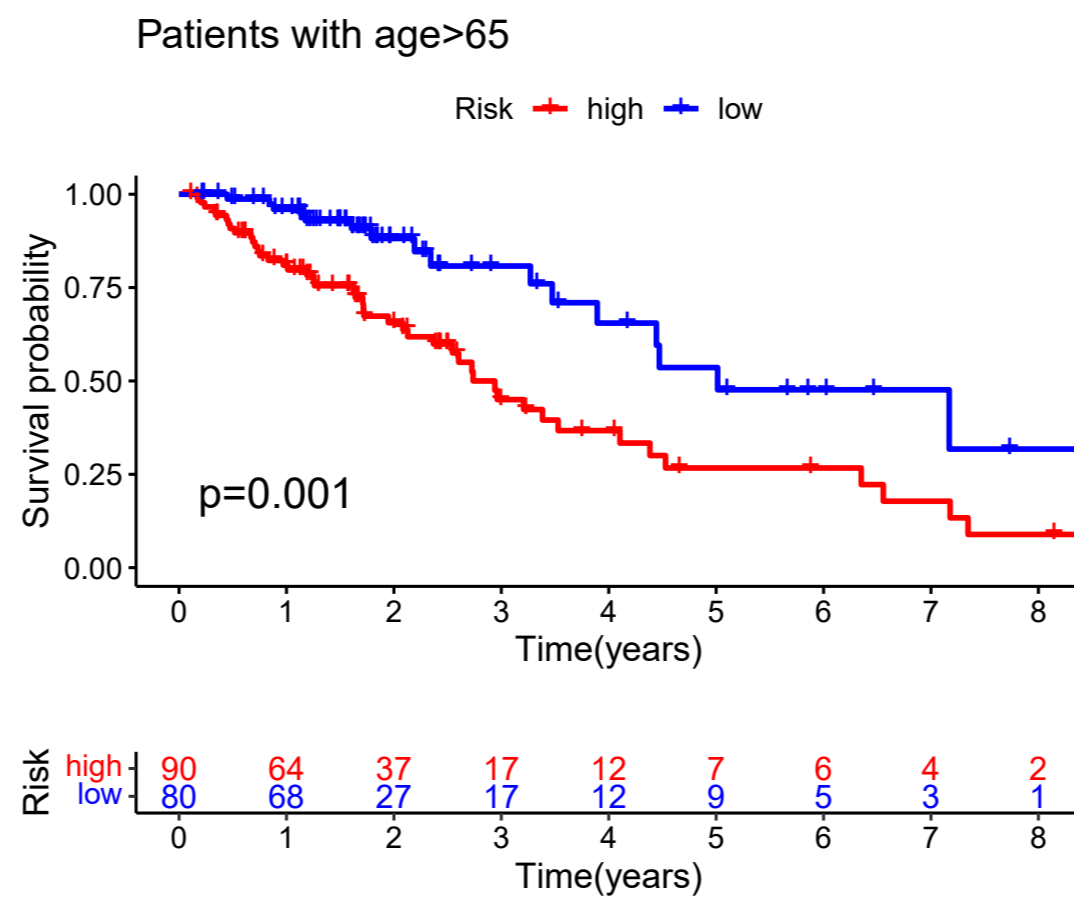

C

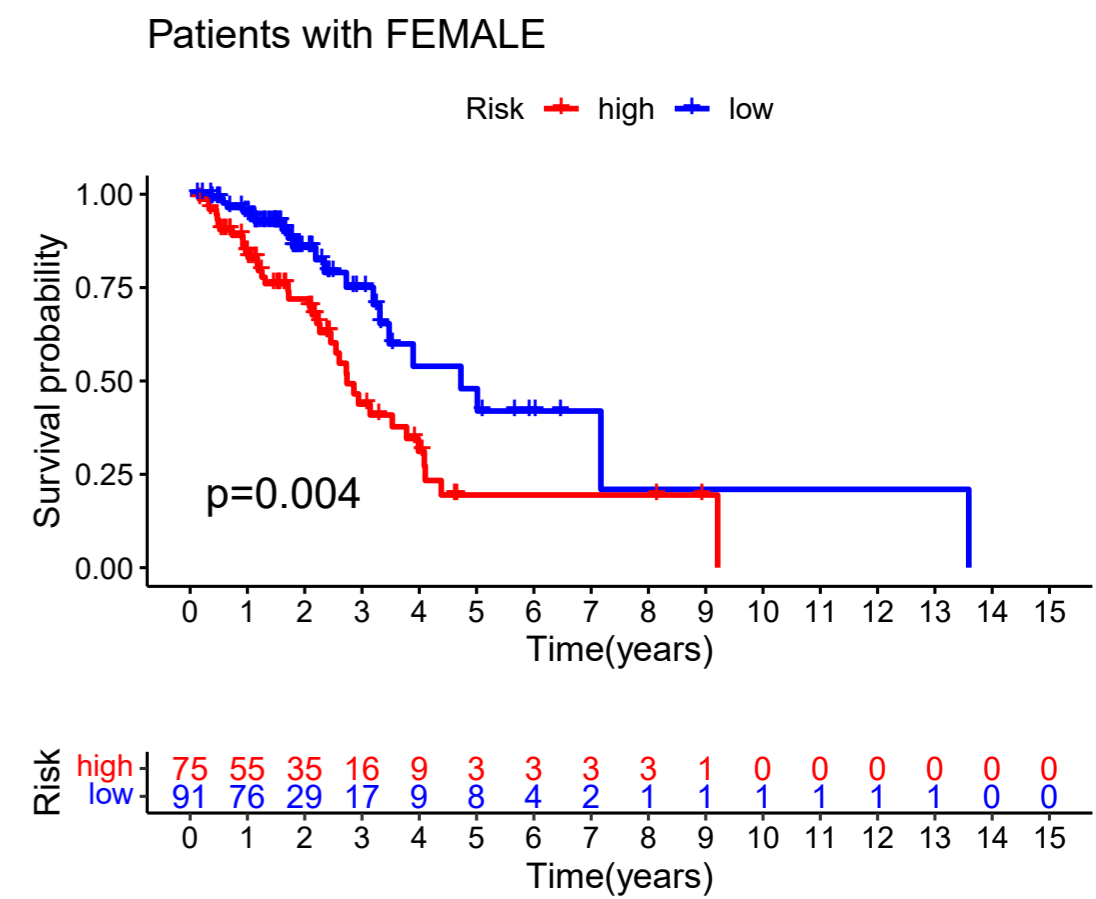

D

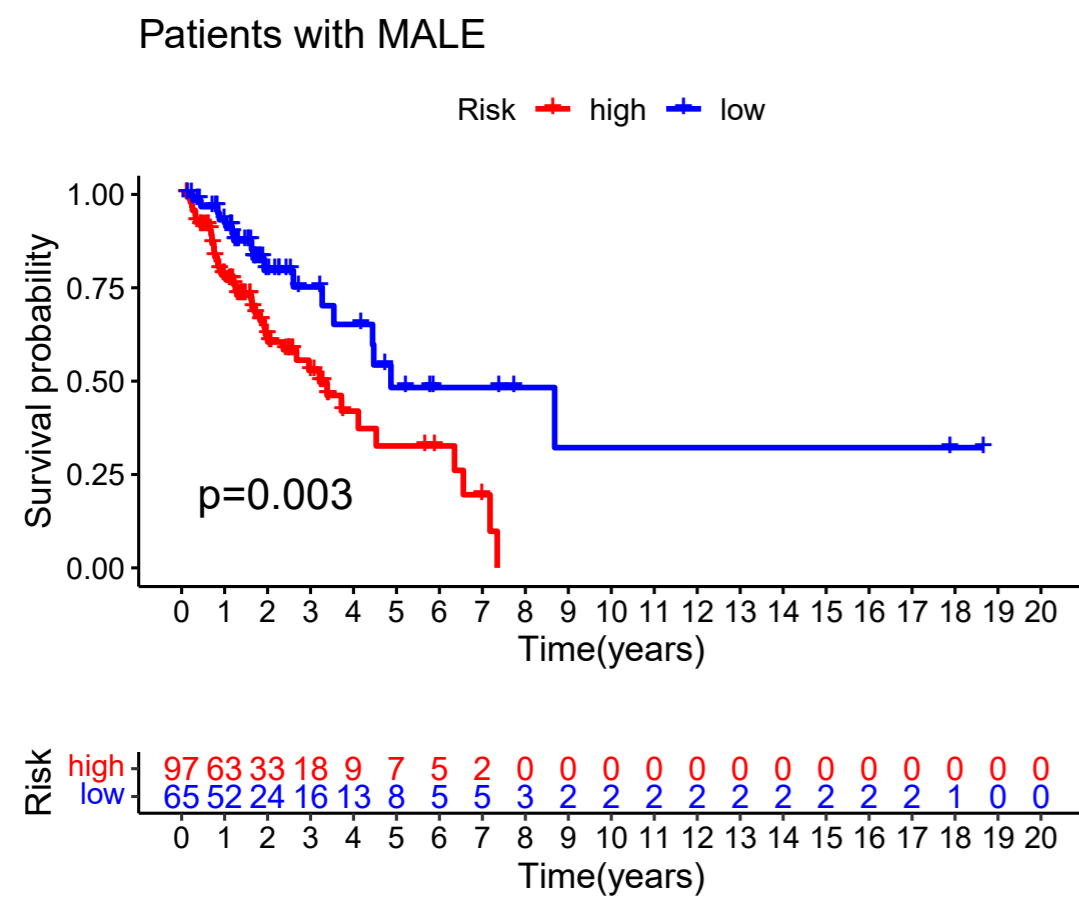

# E

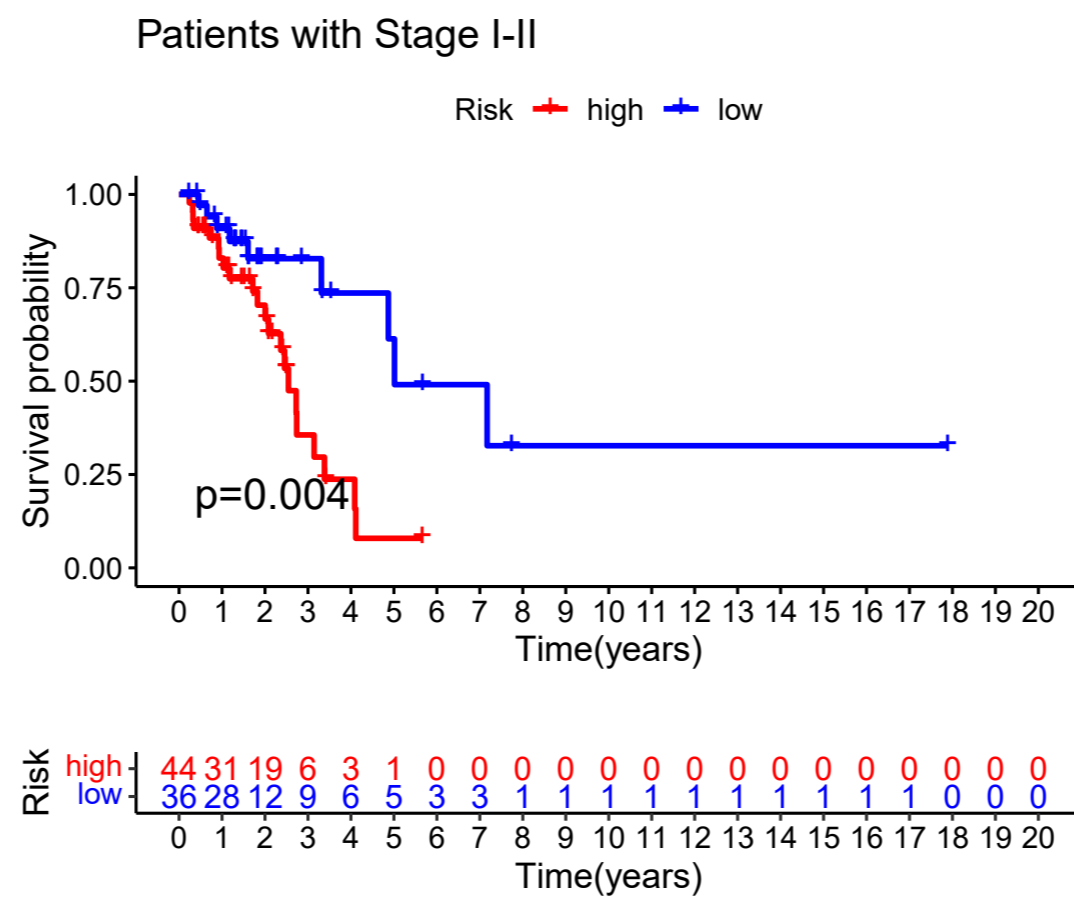

# F

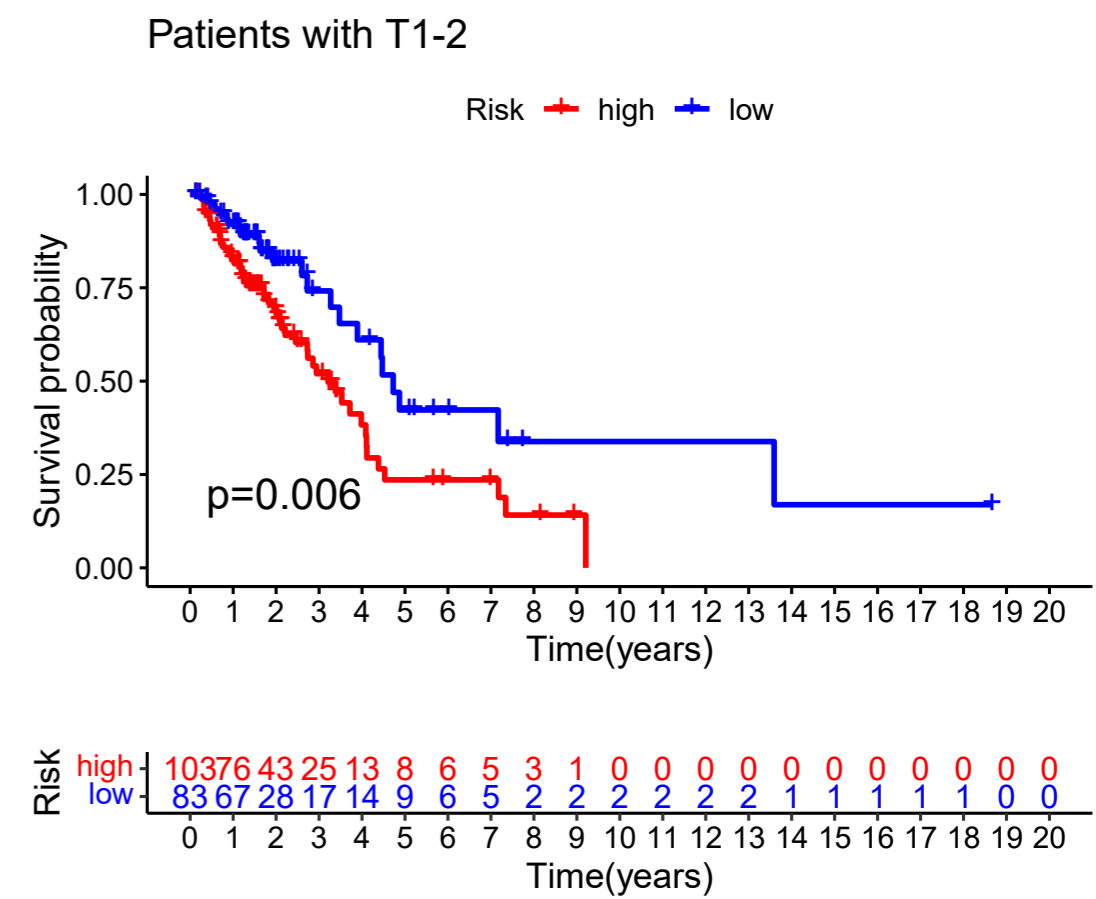

G

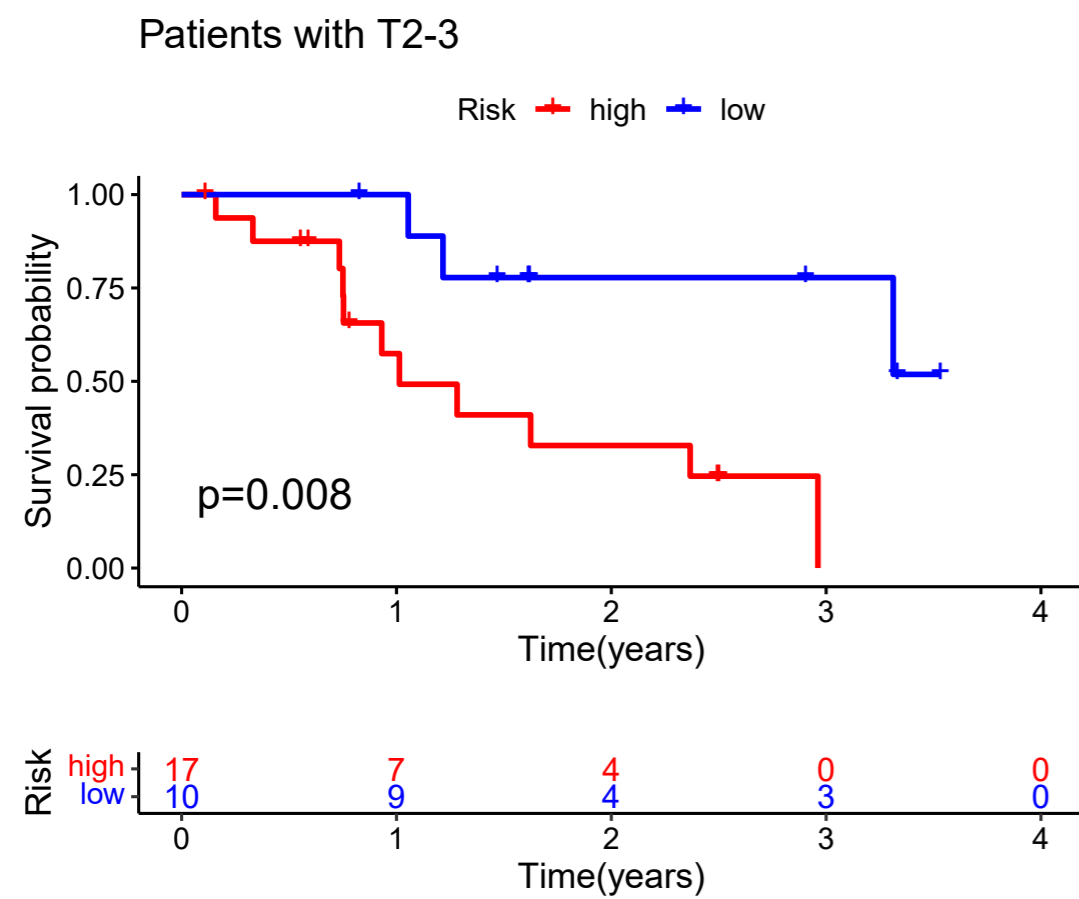

# H

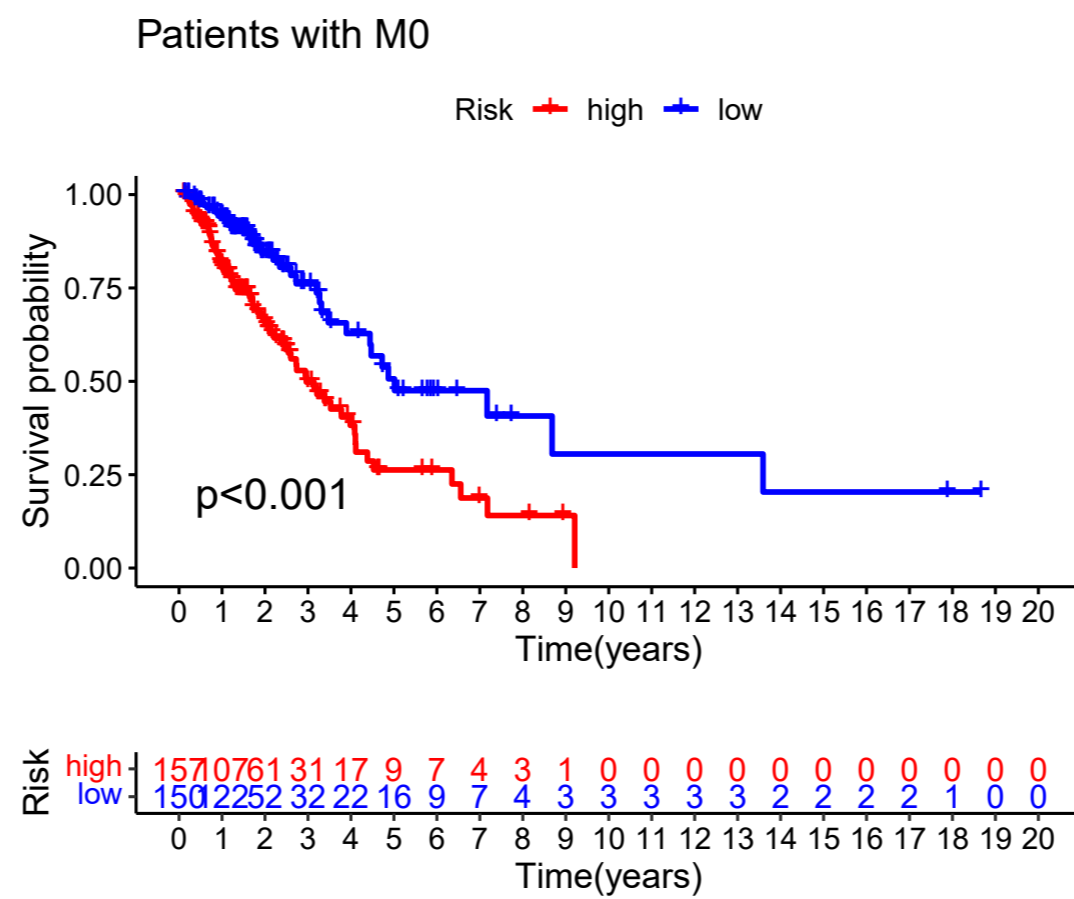

# I

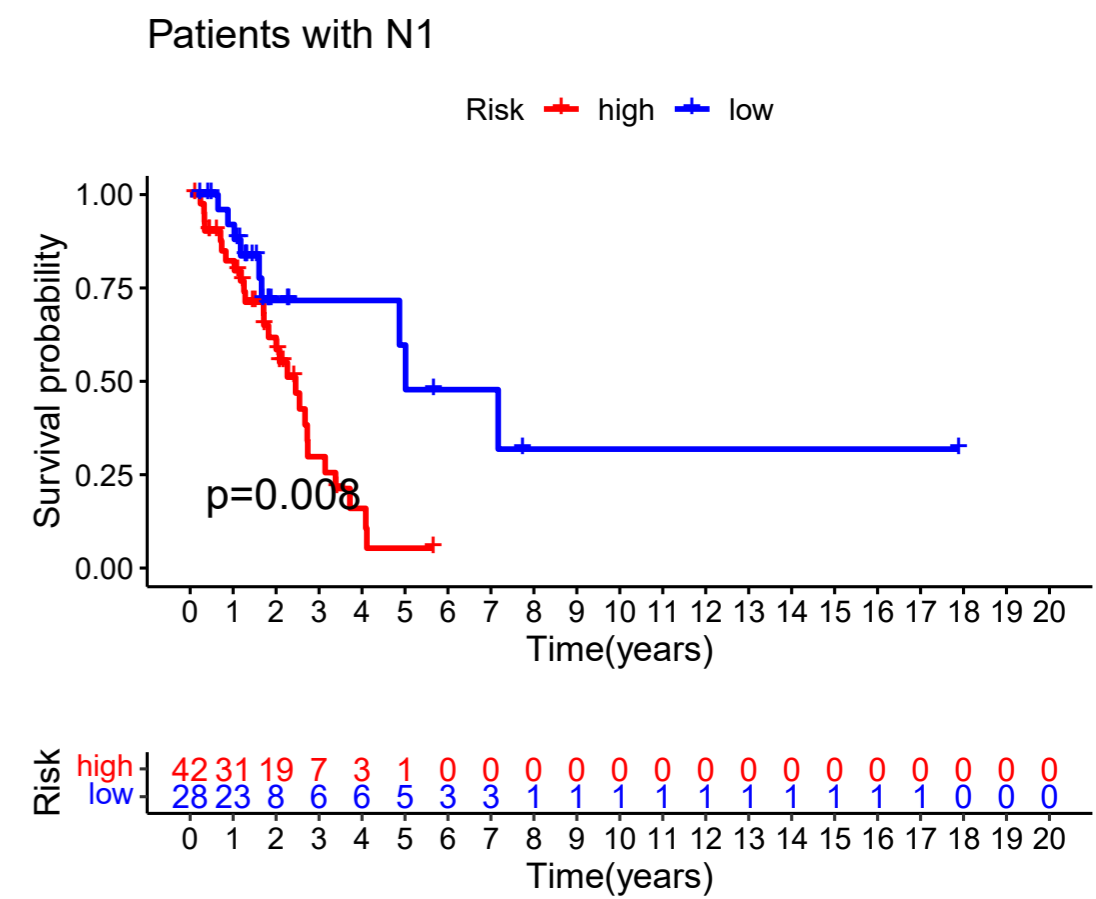

Supplement: Supplementary file 3 — Supplementary Figure S3. [file 41598_2024_57910_MOESM3_ESM.pdf]
