# Supplementary material for: Identification of molecular subtypes and a prognostic signature based on m6A/m5C/m1A-related genes in lung adenocarcinoma
Source: Sci Rep. 2024 Mar 30;14:7543. doi: 10.1038/s41598-024-57910-5 (PMC10981664; doi:10.1038/s41598-024-57910-5)
Supplement: Supplementary file 4 — Supplementary Legends. [file 41598_2024_57910_MOESM4_ESM.docx]

**Supplementary Information**

Supplementary Figure S1: The study flowchart.

Supplementary Figure S2: The histograms show the read count distributions for all 9 genes associated with prognosis: (A) *TNS1*, (B) *SNHG12*, (C) *PABPC1*, (D) *IGF2BP1*, (E) *FOXM1*, (F) *CCNB1*, (G) *IGF2BP3*, (H) *CBFA2T3*, and (I) *CASC8*.

Supplementary Figure S3: The low-risk group had a better prognosis than the high-risk group in stratification analysis based on the clinicopathological parameters, such as age (a, b), sex (c, d), stage (e), T stage (f, g), M stage (h), and N stage (i).

Table S1: The high-risk group enriched gene sets.

Table S2: Gene sets for immune cells or immune fractions.

Table S3: Gene sets for stemness, mesenchymal EMT, tumorigenic cytokine, and angiogenic activity scores.
